# Using the stages of change model to develop an understanding of caregiver intent for family behavior change following pediatric testing for genetic obesity: A qualitative analysis

**DOI:** 10.1016/j.obpill.2025.100227

**Published:** 2025-11-08

**Authors:** Eileen Chaves, Emily Kunkler

**Affiliations:** a Psychology & Neuropsychology, The Ohio State University of College of Medicine, Center for Healthy Weight and Nutrition, Nationwide Children's Hospital, 700 Children's Drive LAC, Suite 5F, Columbus, OH, 43215, USA; b Center for Healthy Weight and Nutrition, Nationwide Children's Hospital, 700 Children's Drive LAC, Suite 5F, Columbus, OH, 43215, USA

**Keywords:** Genetic testing, Childhood obesity, Caregiver experience, Qualitative research

## Abstract

**Introduction:**

Obesity rates continue to rise in children and adolescents worldwide. The heritability of obesity is estimated to be 40–80 %. Genetic testing for monogenic causes of obesity in the clinical setting is increasing; however, how results of these tests affect family behaviors is unclear. The objective of this study was to understand caregiver intent to change family behaviors following genetic testing for obesity.

**Methods:**

The sample from this qualitative analysis derives from a larger study identifying mutations on the melanocortin 4 receptor (MC4R) pathway. Inclusion criteria included participation in the main study, aged 2–17 years, and a history of severe obesity and hyperphagia. Caregiver-child dyads were recruited to ensure equal representation of genetic results across racial/ethnic subgroups (Non-Hispanic White, Black, Hispanic). A third of participants in the main study enrolled in the sub-study. Structured caregiver interviews were analyzed using grounded theory.

**Results:**

Twenty caregivers were female, 55 % White, 45 % Black, and 5 % Hispanic. Mean caregiver age was 42.3 ± 6.5 years and BMI 40.5 ± 7.9 kg/m^2^. Majority of children were male (55 %), mean age was 10.0 ± 4.7 years and BMI 40.8 ± 9.9 kg/m^2^. Interview themes related to all stages except for maintenance stage in the Stages of Change Model (SoC). After receiving their child's genetic test results, regardless of outcome, the majority of caregivers in the study reported either remaining in the contemplation, preparation, or action stage or were motivated to move forward to a more action-oriented stage.

**Conclusions:**

Genetic testing for pediatric obesity, regardless of test outcome, motivates caregivers to move forward within the SoC Model or to remain in their current stage. These findings suggest that engaging caregivers to have their child with obesity tested for a genetic cause of obesity does not cause families to stop engaging in behavior change, unless the family was not ready to engage in behavior change prior to genetic testing.

## Key Takeaways

• Genetic testing may aid diagnosis and serve as a therapeutic tool to boost caregiver understanding and engagement.
• Family-centered counseling approaches are essential when discussing testing to ensure trust and collaboration.
• Clinicians should assess caregiver readiness and tailor messages to match each family's motivations and concerns.

## Graphical abstract

**Figure:**
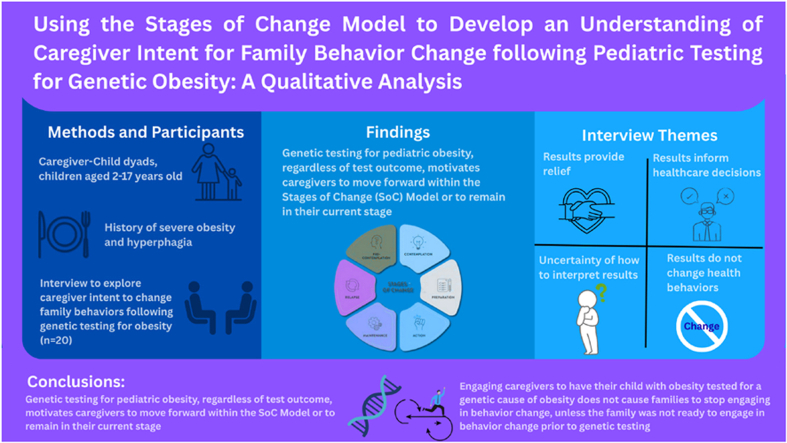

## 1 Introduction

Studies suggest genes account for 40–80 % of the risk for obesity [1]. The presence of certain genetic variants or mutations can lead to increased hunger levels, reduced satiety, and a tendency to store body fat [2]. Genetic causes of obesity can be either monogenic, such as on the melanocortin-4 receptor (MCR4) defect; syndromic, such as Prader Willi syndrome; or polygenic, a cumulative contribution of several genes amplified in an obesogenic environment [3].

The current emphasis in the field of obesity medicine is to better understand the polygenic factors that may underlie susceptibility to obesity and/or predict response to treatment [4,5]. This new direction aligns with the concept of precision medicine [6,7] and provides an opportunity for innovation and breakthrough with obesity treatment. However, there are concerns about how individuals may understand obesity-related genetic tests or perceive their risk for obesity and how these results may influence lifestyle behavior and treatment choices. In addition, the level of certainty or uncertainty between a genetic variant or mutation and risk for obesity further complicates how individuals understand or relate to the results. For example, a result interpreted as a variant of unknown significance (VUS) or a heterozygous or homozygous genetic variant or mutation for obesity introduces a complexity in predictive risk that may be challenging for providers to discuss or for most patients to understand. Even when the genetic test result is understood, a negative or positive result can have divergent implications. While some individuals may disengage from attempts at treatment and develop a more fatalistic outlook, others may seek to intentionally address modifiable risk factors [8,9]. Furthermore, what individuals expect from genetic test results may also affect how they respond to the results [10]. For example, individuals with a family history of obesity may expect that genetic expectations translate to behavior change if the expectations are either confirmed or disproved.

To date, there are no studies on parental understanding or perception of polygenic test results for obesity in children. Existing studies conducted in adults have had mixed results [11,12]. Some studies found a positive test on genetic susceptibility to obesity did not adversely affect confidence in ability to lose weight or control eating behavior and led to a decrease in self-blame [12]. Conversely, negative testing or having a lower perception of genetic risk for obesity has led to decreased motivation to implement lifestyle behaviors to overcome genetic susceptibility [11].

Qualitative studies exploring parental experiences related to their child's genetic testing results for other conditions, e.g., developmental delays, report parental challenges with understanding the genetic results, especially when they are variants of unknown significance (VUS) [13,14]. Polygenic testing related to obesity is an emerging field, so although a number of genes may be associated with obesity, definitive studies are lacking that demonstrate a causal relationship. When undergoing genetic testing for susceptibility to obesity, results include VUS, thus limiting definitive knowledge about the predictive risk. Investigating how parents/caregivers grapple with the uncertainty surrounding these types of results or their implications for treatment decisions is vital.

The purpose of this study is to understand what factors underlie parental/caregiver decisions to have their child tested for genetic susceptibility for obesity and how the genetic results influence parental/caregiver understanding and decision-making related to weight management and lifestyle behaviors.

## 2 Methods

### 2.1 Design and recruitment

This qualitative analysis is an extension of a larger multi-site study examining potential genetic causes of obesity across pediatric and adult participants within the Center for Healthy Weight and Nutrition (CHWN) at Nationwide Children's Hospital [15]. Structured interviews and validated surveys were employed to contextualize the experiences of twenty caregivers of children and youth previously tested for the presence of a genetic mutation in the melanocortin-4 receptor (MC4R) [13]. Data collection occurred from September 2020–August 2021 with approval from the institutional review board at Nationwide Children's Hospital.

Eligible participants in the original genetic obesity study were contacted via phone regarding an opportunity to participate in an additional study in relation to their child's prior genetic testing. Inclusion criteria included the ability to read and speak English and participation in the primary genetic obesity study. Inclusion criteria for the original study included individuals aged two and older with a history of severe obesity or early-onset obesity, as well as a history of hyperphagia. For children aged 2–17 years old, severe obesity is defined as 1.4 times the 95th percentile of age and sex-specific body mass index [16]. A 13-item survey was used to determine the presence of hyperphagia [17]. If caregivers were interested in being included in the study, they were provided with a document outlining a description of the project and how their anonymized data would be collected and used, as well as completing an online consent form via RedCap. Exclusion criteria for the original study included a diagnosis of Prader-Willi syndrome, craniopharyngioma, or surgical procedure in the hypothalamic region. Twenty caregiver-child dyads, with 10 index children with a caregiver in each group, were recruited using convenience sampling to ensure equal representation of genetic results across racial/ethnic subgroups.

### 2.2 Data collection

Demographic information, family history of obesity and related complications, and weight history were obtained using questions from the National Health and Nutrition Examination Survey (NHANES). The 20-question Food Nutrition and Physical Activity (FNPA) survey was used to measure the child's diet and physical activity behaviors [18]. Additionally, family functioning was assessed using the McMaster Family Assessment Device (FAD) – General Functioning (GF) subscale [19]. Parental weight and height measures were obtained and categorized using body mass index (BMI) into normal weight (BMI 18.5-24.9), overweight (BMI 25-29.9), and obesity (BMI≥30) [20,21].

An interview guide was created according to the initial study aims. Questions were developed reflecting themes from existing literature and based on the Health Belief Model [22,23]. The final interview guide followed a semi-structured format and included primarily open-ended questions [See Appendix A].

Two investigators conducted 20 caregiver interviews via an online video platform with an average length of 55 min. Researchers determined content saturation when redundancy of data and a lack of new emerging themes during the final few interviews were reached.

### 2.3 Analysis

All interviews were audio-recorded on the Webex video platform, transcribed via a transcription service, and then uploaded to Dedoose v8 software for qualitative analysis [24]. Three researchers inductively coded all interviews independently according to grounded theory methodology using the constant comparative method [25,26]. Reviewers engaged in collaborative discussion over three separate meetings to converge independent codes into a smaller number of codes and emergent themes.

Through continuous discussion, researchers further reconciled emergent themes to determine their relation to the Transtheoretical Model of behavior change, or the Stages of Change Model (SoC) [27]. Each theme was examined by all three reviewers for its direction of movement along the SoC model: forward, stagnant, or backward.

## 3 Results

### 3.1 Participants

All caregivers were female and were the primary caregiver of the child tested. Nearly all caregivers were a parent, with the exception of one caregiver who was the adoptive mother of the child. Because of this, one dyad in the study was not biologically related. Mean caregiver age was 42.3 ± 6.5 years and BMI 40.5 ± 7.9 kg/m^2^. Majority of children were male (55 %), mean age was 10.0 ± 4.7 years and all had BMI-for-age >95th percentile.

The mean FNPA score was 55.4 ± 6.4 on a scale of 4–80. Higher scores indicate more positive nutrition and physical activity behaviors. Most caregivers (n = 14, 70 %) reported non-problematic family functioning per FAD scores. Six caregivers (30 %) reported problematic family functioning, indicating poorer family functioning. Participant characteristics are summarized in Table 1.

**Table 1:** Participant characteristics.

**1.1 Caregiver Characteristics**	

**Caregivers**	

**Sex** %, (n)	**Female** 100 (20)
**Race/Ethnicity** %, (n)	**Non-Hispanic White** 55 (11)
	**Non-Hispanic Black** 45 (9)
	**Hispanic** 5 (1)
**Age**, Mean ± SD	42.3 ± 6.5 years
**BMI**, Mean ± SD	40.5 ± 7.9 kg/m^2^

**1.2 Child Characteristics**	

**Children**	
**Sex** %, (n)	**Male** 55 (11)
	**Female** 45 (9)
**Race/Ethnicity** %, (n)	**Non-Hispanic White** 55 (11)
	**Non-Hispanic Black** 45 (9)
	**Hispanic** 5 (1)
**BMI**, Mean ± SD	40.8 ± 9.9 kg/m^2^

**1.3 Household Characteristics**	

**Household**	
**FNPA Score**^a^, Mean ± SD	55.4 ± 6.4
**Family Functioning**^b^, % (n)	**Non-Problematic** 70 (14)
	**Problematic** 30 (6)

FNPA = Food and Nutrition Physical Activity.

a Higher score indicates more positive nutrition and physical activity behaviors, 4-80.

b McMaster Family Assessment Device.

### 3.2 Preliminary themes

**Fig. 1:**
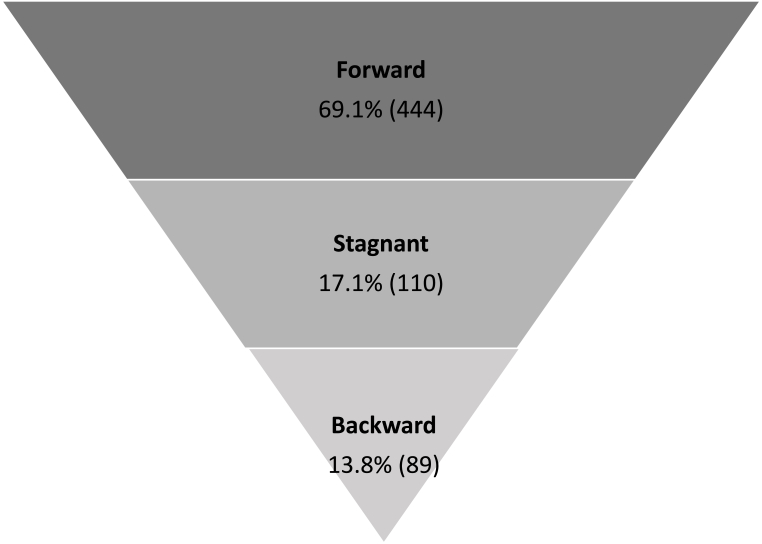
Quote distribution among the stages of change model, % (n) (n = 643).

Themes promoting caregivers and their families moving forward along the SoC model include 1) results provide relief, 2) results inform healthcare decisions, and 3) solace in knowing results benefit others. Themes having no effect on the current stage of change include 1) uncertainty of how to interpret test results and 2) results do not change current health behaviors. One theme was determined to promote regression in the current stage of change: results are discouraging for the child's current treatment plan. Theme descriptions and exemplary codes can be found in Table 2. Each theme's effect on the movement through the SoC and exemplary quotes can be found in Table 3. Distribution of quotes within the SoC model can be found in Fig. 1.

**Table 2:** Qualitative themes.

Movement Along SoC Model	Theme	Definition	Exemplary Codes
**Forward**	**Results provide relief**	Genetic test results provided relief, hope, answers, and next steps for some participants. Parents expressed feeling alleviated of guilt over child's condition (positive test result).	Genetic testing gives answers Genetic testing gives relief Alleviation of parental guilt due to genetic test results
**Forward**	**Results inform healthcare decisions**	Genetic test results were empowering and motivated healthcare decision-making, including medical management, physical activity, and family lifestyle changes.	Genetic result inform care Genetic testing informs health decisions Genetic test result is empowering
**Forward**	**Solace in knowing results benefit others**	Participants expressed participation in study allowed them to make a positive difference for others and the future of obesity medicine.	Altruism Helping others through result Research benefitting the future
**Stagnant**	**Uncertainty of how to interpret test results**	Participants were uncertain of how genetic test results affected their current treatment plan.	Results feel neutral Present implications of test results are limited Uncertainty of what test results mean
**Stagnant**	**Results do not change current health behaviors**	Genetic test results did not affect current health behaviors such as physical activity and dietary intervention.	Results make no difference Genetic result does not change actions Genetic result does not change treatment plan
**Backward**	**Result is discouraging for current treatment plan**	Participants expressed frustration or emotion about genetic test results, expecting validation for current treatment plan or clearer direction for a modified treatment plan.	Frustration with lack of next steps Emotion about negative test result Positive result is discouraging for treatment plan

**Table 3:** Theme representative quotes.

**Theme**	**Child Ethnicity + Genetic Results**	**Caregiver Characteristics**	**Representative Quote**
**Forward**			
**Results provide relief**	Non-Hispanic	Non-Hispanic	Mentally, I thought maybe **it would help settle** like that **dispute as a parent**. I have in my mind … **she can't ever have any fast food**, we can't ever have any cake, and **then when you see that it doesn't affect** … **everyone** in the family **the same way** and it just affects her more, **it's more of like it's a sigh of relief**, like it's something **more than what I'm feeding her**. I know it's not good always, you know, but I'm just saying, it's kind of like, okay, **I'm not crazy that it's just what I'm feeding her.** Because **we can eat salad every day**, and **she would be the one to gain** and no one else would.
	Black	Black Female	
	Positive	39 yrs	
**Results inform healthcare decisions**	Non-Hispanic	Non-Hispanic	Me and her sit around and talked … **whatever [the genetic result] comes back, we'll choose that path and we'll just figure out a way around it**, you know, **because if it's genetic, we know there's nothing you can do about it**, **but if it's not genetic, there's a lot you can do about it.** There's a lot of different choices you can make, a lot of different actions you can make.
	White	White Female	
	Negative	44 yrs	
**Solace in knowing results benefit others**	Non-Hispanic	Non-Hispanic	This study is important to be a part of because **now I can have more curiosity and more movement towards true solutions to this problem, not just for me, but for other people.**
	White	White Female	
	Positive	50 yrs	
**Stagnant**			
**Uncertainty of how to interpret test results**	Non-Hispanic	Non-Hispanic	**We don't know what that positive [genetic result] means**. We don't have so much information to know, **and if I take any other type of drastic measures, what does that mean for … whatever this is that could be** something else. **I'm really not sure how it's going to affect moving forward.**
	Black	White Female	
	Positive	39 yrs	
**Results do not change current health behaviors**	Non-Hispanic	Non-Hispanic	I mean we always know, **we know our [lifestyle] choices and what's right and what's wrong** and knowing that we should be drinking more water and walking more and exercising more for good health, maybe not even just for weight loss but just for good health, so no, **I don't feel like it's going to change it very much.**
	White	White Female	
	Negative	40 yrs	
**Backward**			
**Result is discouraging for current treatment plan**	Non-Hispanic	Non-Hispanic	Well, then **[the genetic test result] makes me even more worried because now I know he's got this that he's going to have to battle [it] forever.** You know, **he's going to have to work harder** because I know what that's like [with] me having thyroid. It's just nothing seems to work, so I feel like **he's got to work harder than other people now because he's got [a positive result]** – and **I also don't want him saying, "Well, I have this genetic problem, and that's why I'm big."** You can't do that. You know, **you can't sit back and just say, "I'm big because of this," and let it go.** You know, **you got to work harder than other people, unfortunately.**
	White	White Female	
	Positive	48 yrs

#### 3.2.1 Forward theme

##### 3.2.1.1 Results provide relief

Many caregivers reflected on how their child's test results allowed them to pinpoint a potential cause of their child's weight. For dyads that received positive results, some caregivers reported relief from guilt from potentially playing a role in their child's weight gain:

PARTICIPANT: … it makes me feel a little bit better because now I don't feel like I have to blame myself for it, all of it, you know what I mean?

INTERVIEWER 1: Mm-hmm. Do you feel like in the past, Mom, you kind of did take some of that blame on?

PARTICIPANT: **I did. I did. I took all of it, every bit of it.** (Participant 22).

Among dyads with older adolescents, caregivers reported that a positive result also provided relief to the child, with one parent saying,

“Well, I think for him, and well, for myself as well, it's confirmation what I already knew, but benefit for him is like, you know, **this is not your fault**. And that's the problem with being heavy is that everybody tells you it's your fault all the time, you're not doing something that you should be doing, and if you did this, then this would be the result, and it is just not true. It's just not.” (Participant 2).

Among dyads that received negative results, caregivers frequently touted that the results reinforced their belief that sustained lifestyle changes could have the greatest impact on their child's weight:

“**We have to make changes** because there's nothing like, you know, genetic or hereditary or anything like that. So anything that's not hereditary or, you know, genetic, **you can make the changes and you can fight it**.” (Participant 13).

Some caregivers reported that the negative result gave both them and their child more confidence moving forward that their current health efforts were worthwhile:

“It's not a clue anymore where we didn't know. We know now. **We have the results, so we can move forward**. We can make changes. We can start eating healthy. We can get up and start moving more. You know, if we – each different thing plays a factor in weight gain and weight loss because if you don't do it, you're going to gain the weight. If you do it and stay consistent, the weight's going to stay off. You're going to be healthier, and [my daughter], she knows that.” (Participant 18).

##### 3.2.1.2 Results inform health decisions

Another common theme among caregiver interviews was that results helped to inform future health decisions. Some caregivers reported that they felt better equipped to make decisions on behalf of their child after learning their test results. One caregiver reported,

“The benefit I was thinking was, you know, if it was genetic, then maybe there's a different path that we needed to go down. You know, I don't know, like if something in the body wasn't working right, you know, then **maybe there's another medical path that we were supposed to go down**.” (Participant 19).

Depending on the age of the child and their involvement in testing, some caregivers reported opting to undergo genetic testing empowered the child to be more involved in their health, reinforcing current behaviors or promoting them to initiate new health-promoting behaviors:

“I would say I was shocked in the way I was surprised that it was negative. Just seeing [my daughter] gain the rapid weight gain, she ended up having severe sleep apnea behind all the weight gain. She was on borderline diabetes. She changed that. She changed that. **She said no, she wasn't allowing that, so [my daughter], that's when she really got serious with it and started really understanding what was going on with her body** that weight had to come off.” (Participant 18).

##### 3.2.1.3 Solace in knowing results benefit others

Many caregivers discussed altruism as either an initial motivator or a “bonus” outcome of having their child participate in the primary study for genetic testing:

“I was like that's what made me decide yes, go ahead and draw, you know, **anything that you can do that will help him** at the end of the result **and help other people** out there that want to make use of it.” (Participant 20).

“I just felt like, I mean, **what did we have to lose?** You know, the only thing we could do was gain information, and like I said, it was either going to include him or it was going to exclude him as something that, you know, didn't apply to him at all. So to me, it's better to go into a situation and gain full knowledge than you just have a piece of the puzzle. So, you know, as more science comes out, **if it's something that can benefit others, you know, what's the harm?** There wasn't anything that he was going to be hurt doing. You know, we weren't putting him through any kind of strenuous tests, or anything like that, so I don't feel that anything bad could have come out of it, only good information, the **knowledge to move forward**.” (Participant 3).

Caregivers commonly referenced family or personal experiences with weight, stating that they were happy to “pay it forward” both to help inform future best practices as well as to reduce future stigma related to overweight and obesity:

“I mean just like it's a subject that I'm really passionate about, so if there's anything that I could do because it's affected me. **I lived it and breathed it and still do, so anything I can do to help, I would do it because it's an important cause to me**. You know, it's something that I'm passionate about, so I knew you said you had this genetic testing and you're doing a study, well, absolutely because anything I can do to help, I'm all for it.” (Participant 9).

Despite the limited existing research on treatment specific to children with various genetic causes of obesity, caregivers expressed that they took comfort in knowing that their participation in the main research study may benefit future generations of children:

“I didn't know, you know, what does that mean for us, so I didn't really think much of it, to be honest with you, just because I know we're still in the early stages of all of this and I don't know that the dots will be connected until maybe he's an adult. You know, I'm not sure how that will look, but I know **I just wanted to help and I'm glad that we were able to provide information** to look further into.” (Participant 3).

#### 3.2.2 Stagnant theme

##### 3.2.2.1 Uncertainty of how to interpret test results

Despite the results of the genetic testing, caregivers frequently expressed that they were unsure of how to understand the meaning of their child's results. One parent stated,

“I just thought I would know more. I don't know, I felt like I would just … **I wanted more answers**, I guess.” (Participant 16).

“Well, I thought it was a good idea. The only thing I guess I didn't like or whatever was her results came back and kind of like, **we don't really know what's wrong**. Like, I thought it was going to be, like, “Yay, we'll know something” but it was kind of like we didn't. Hers was kind of like a rare one. So, yeah, I mean I liked it. I liked the idea.” (Participant 16).

“So I think that would be my hesitation, as I wouldn't want to, you know, take those kind of measures [metabolic bariatric surgery] and here **there's something underlying that's really the cause**, or the root, or something that's going to still push his weight back up again.” (Participant 19).

A few caregivers even had trouble recalling their child's results, indicating that it likely had little effect on family behaviors.

##### 3.2.2.2 Results do not change current health behaviors

For some caregivers, the genetic test results had little influence on their current lifestyle or treatment plan. As one caregiver stated:

“I think we were already on the mindset of, regardless of what was going on, we all needed to be healthy, we all needed to do better. So **it didn't necessarily really change it, getting the results**.” (Participant 4).

“I mean we always know, we know our choices and what's right and what's wrong and knowing that we should be drinking more water and walking more and exercising more for good health, maybe not even just for weight loss but just for good health, so no, **I don't feel like it's going to change it very much**.” (Participant 9).

This perspective was observed across dyads despite their genetic test results, particularly by those who reported higher active engagement with healthy lifestyle behaviors prior to testing. Notably, while caregivers whose child received a positive result expressed that it helped them to be less restrictive toward their child's behaviors, several also emphasized that they did not interpret the result to be an excuse to give up on efforts to manage their child's weight:

“I don't want it to be like, "Well, I'm this way because I have this genetic thing." You know, it's not an excuse. It's not a crutch, but at the same time, **it's a reason to adapt what you're doing to try to address the issues that need to be addressed. It's not an excuse**.” (Participant 28).

#### 3.2.3 Backward theme

##### 3.2.3.1 Results are discouraging for current treatment plan

Some caregivers—particularly those who had received positive results with little clinical implications—stated frustration with how their child's test results provided no new information to inform their current treatment plan. One parent stated,

“Now, what was very disappointing to me, and really quite upsetting to me, actually, is, so we got the call about his results, that he does have an abnormality, and we were meeting with the doctor regarding that, so we went to the appointment, and basically, it's like, yes, he does have an abnormality and there's nothing we can do about it. **So there was no next steps.** There was next steps for people who had a different abnormality. So I think that was almost like, if I would have known the answer was he has an abnormality and there's nothing we can do about it, I would have gone for the consult about it after. Like that was a waste of my time. That's what I felt.” (Participant 2).

The confusion some caregivers expressed after receiving their child's result—whether positive or negative—sparked feelings of parental guilt and helplessness about how to help manage their child's weight moving forward. Regarding the negative result, one parent confided,

“Oh, I was [devastated]. I was really – **I really just wanted to be able to explain it away** like, "Oh, yeah, he's got a chemical imbalance in his brain, and he's always not being ever full," and **it was just easier** to, you know, tell people that than say, "Oh, no, I was a young mom, and I messed up, and I overfed my kid, and then he became obsessed with food, and we're no good for each other." (Participant 17).

## 4 Discussion

This study explored caregiver reactions to pediatric genetic testing for obesity and its impact on motivation for health-related behavior change. The findings suggest that, regardless of the test result, genetic testing may encourage most caregivers to move forward or maintain their current position in the SoC Model. Undergoing genetic testing did not result in a regression to earlier stages of change, nor did it lessen caregiver engagement in family-based pediatric obesity treatment when compared to baseline.

The results of this study challenge the idea that identifying a genetic cause for obesity may reduce perceived control or motivation to engage in behavioral interventions for obesity treatment. Instead, genetic testing appears to reinforce or sustain caregiver motivation, perhaps by offering a biological explanation that can relieve guilt or reduce stigma, while simultaneously validating the need for continued intervention. This reframing of obesity as a multifactorial condition may promote a more balanced perspective and engagement in care.

Furthermore, these findings suggest that genetic testing, independent of testing result, can serve as an empowering tool that enhances family health autonomy. Providing families with tangible, personalized health information may cultivate a sense of agency, promote collaborative treatment planning, and encourage continued engagement in care.

Of note, no significant differences were seen between themes among caregivers of different races/ethnicities, although this was reviewed specifically as a parameter for content saturation. Additionally, the impact of the child or youth's age at the time of the genetic test result on the caregiver's understanding or perception of the result and subsequent family behavior change was not explored as a priority in this study.

Understanding caregiver perceptions and expectations regarding genetic testing is critical to effective clinical counseling and shared decision-making. Clinicians should approach genetic testing conversations with empathy and openness, recognizing the nuanced ways stigma may influence family readiness for change and decision-making. These insights can inform the development of structured shared decision-making aids and clinical pathways, ensuring that discussions about testing are framed appropriately, ethically, and supportively.

Further directions for this research include exploring how genetic test outcomes for obesity (e.g., positive vs. negative findings) influence long-term caregiver attitudes and family behavior change trajectories. Understanding how children and youth perceive their own genetic test results is also important for providing a better understanding of how this impacts not only their individual attitudes and behavior over time, but also the behavior of their families. Qualitative studies may help enhance understanding of the role of stigma plays in decision-making around genetic testing. Additionally, longitudinal research is needed to examine whether genetic testing impacts health behaviors or weight outcomes over time.

## 5 Limitations

The authors acknowledge several limitations when interpreting the findings of this study. The sample size was relatively small and recruited from a single institution, which may limit the generalizability of the findings to broader or more diverse populations. Although the study incorporated racial and ethnic diversity through purposive sampling, the use of a convenience sample may have introduced selection bias, as participating caregivers may have had higher levels of motivation or differing views on genetic testing compared to those who declined participation.

Additionally, this study focused only on caregiver experiences following monogenic testing for melanocortin-4 receptor (MC4R) mutations, which may not reflect caregiver responses to polygenic testing, a noted area of interest in the field of precision medicine. Given the complexities and uncertainties associated with interpreting polygenic risk scores and variants of unknown significance (VUS), findings from this study may not be fully applicable to families receiving more ambiguous testing results.

The qualitative design provides valuable insights but limits causal inference. The mapping of themes to the SoC Model was interpretive and not based on quantitative assessment, which may affect the precision with which transitions between stages are described. Additionally, caregiver expectations, previous beliefs about obesity, and exposure to stigma may have influenced both the interpretation of test results and caregiver responses during interviews. This could contribute to response bias, particularly in a retrospective, self-reported context.

Another notable limitation is the lack of direct assessment of the child or youth's perspective on the genetic testing process. While caregiver perceptions are central to decision-making in pediatric care, understanding how youth interpret and internalize genetic risk may be equally important in evaluating long-term impacts on both individual and family-level motivation and behavior change.

Finally, all interviews were conducted during the COVID-19 pandemic, when disruptions to daily routines and healthcare access was significantly affected. These contextual factors may have influenced caregiver attitudes toward health behaviors and engagement in obesity treatment during the study period.

## 6 Conclusions

Irrespective of the results, genetic testing for pediatric obesity serves as a stimulus for caregivers to either advance within the Stages of Change (SoC) Model or maintain their current stage of change.

• Genetic testing functions as a behavioral catalyst, with caregivers reporting that regardless of test outcome, they are motivated to either maintain their current stage of readiness or can advance toward more active behavior change within the Stages of Change (SoC) Model.
• Caregiver readiness for change is crucial, as families who were not prepared to engage in behavior change before receiving a genetic test usually remained unmotivated, highlighting the importance of baseline readiness in influencing outcomes.
• Genetic testing does not discourage behavior change. Significantly, participation in genetic testing for pediatric obesity did not result in families abandoning efforts to change behaviors, lessening concerns that negative or inconclusive results might reduce motivation for behavior change.

## Author contribution

Eileen Chaves served as the principal investigator, developed and implemented the study, conducted the qualitative analysis, and contributed to the writing of the first draft of this manuscript. Emily Kunkler conducted the qualitative and quantitative analyses, contributed to the writing of the first draft of this manuscript, and provided critical revisions to the manuscript. Both authors reviewed and approved the final manuscript.

## Ethical adherence and ethical review

Ethical approval for this study was obtained from the Institutional Review Board (IRB) at Nationwide Children's Hospital in Columbus, OH, reference number: STUDY00001255. Informed consent was obtained from all individual participants included in the study.

## Declaration of Artificial Intelligence (AI) and AI-assisted technologies

Artificial intelligence tools were used solely to assist with grammar correction during the preparation of this manuscript. No AI tools were used for data analysis, interpretation, or content generation. The authors reviewed and edited the content as needed and take full responsibility for the content of the publication.

## Funding

This project was funded by the 10.13039/100017930Academic Pediatric Association Research in Academic Pediatrics Initiative on Diversity (RAPID) and 10.13039/100000062NIDDK.

## Declaration of competing interest

Neither authors, Eileen Chaves nor Emily Kunkler, have any known conflicts of interest.
