# Supplementary material for: Using the stages of change model to develop an understanding of caregiver intent for family behavior change following pediatric testing for genetic obesity: A qualitative analysis
Source: Obes Pillars. 2025 Nov 8;16:100227. doi: 10.1016/j.obpill.2025.100227 (PMC12657336; doi:10.1016/j.obpill.2025.100227)
Supplement: Multimedia component 1 [file mmc1.docx]

Appendix A

Moderator’s Guide

The Meaning of Genetic Obesity: Parental Perception of Genetic Testing for Children with Obesity (Qualitative Addition to GO-ID Study)

Notes to interviewer:

Test the audio recorder prior to starting the interview as needed. Make sure the recorder is recording, that it has adequate storage space, and the batteries are fully charged. However, If the parent has chosen a Skype interview, this will not be needed as recording will occur directly through Skype.

Begin introduction to the interview, which will be conducted either via phone or Skype, given parent participant preference. Ask parent permission to audio record and transcribe the interview. This will be the second time parents will be asked for these permissions, as they have been asked when initially contacted to schedule the interview. Inform parents that all data will be de-identified and remain confidential. Have parent participants sign study consent forms.

Start the interview casually, conveying themes, though not necessarily verbatim. The goal is to set the parent at ease before beginning the interview.

***Thank you for agreeing to participate in our study.***

***This interview will take approximately 30-60 minutes. You are able to stop the interview or skip any questions that may make you feel uncomfortable at any time during the interview. During the interview you will be asked a number of questions. There are no right or wrong answers. We are interested in your unique perspective on your child and your family. You know your child best and are the expert about how things go for your family, so we just want to learn more from you.***

***Most importantly – please take your time and feel free to talk about whatever comes to mind with these questions. We just want to know as much as possible and to learn more from you.***

[Begin Interview]

***Okay, let’s get started. Please think back to when you first heard about genetic testing for weight. What did you think about the test? Could you now please tell the story of how you decided to have your child get a genetic test for weight.*** Allow the parent to take as much time as needed to tell their story. Try to interrupt as little as possible.

After the parent has concluded their story, say, ***Now I’d like to ask you a few more questions. Again, please take your time and try to answer each question with as much detail as possible.***

1. **How likely do you think it is that your child’s weight will affect their future?**
   1. **How do you think weight might affect your child?**
   2. **Of the things you just mentioned, which of these things are you most concerned about?**
   3. **Do you or anyone you know have personal experiences with weight?**
   4. [Prompt] **How has this affected your life or their life? Can you tell me about this?**
   5. **How much does your family feel that your child’s weight is a problem?**
   6. **What does community mean to you?**
   7. Prompt – Some people think of their community as people in their neighborhood or in their **family, friend,** or faith communities**.**
   8. **How much does your community feel like your child’s weight is a problem?**
   9. Prompt - **What can you tell me about this?** [Perceived susceptibility]
2. **How much do you feel your child’s weight will affect their life, both now and in the future?**
   1. **How do you feel your child’s weight affects their life now?**
   2. **How do you think it will affect their life when they are older?**
   3. **How much of an affect has weight had on your life or the lives of people close to you or your child?**
   4. (if parent thinks weight has impacted their life) **How much have your own experiences with weight influenced your decision to have your child tested for a genetic cause of weight?**
   5. **How much have the other experiences of others, like family members, friends, or others in your community, influenced your decision to have your child tested?** [Perceived severity]

1. **What did you expect the test results to be?**
   1. **How did you feel about the results of the test?**
   2. **What possible benefits did you see to having your child genetically tested?**
   3. **What are some possible downsides to having them tested?**
   4. **Does this result change how you think about your child’s weight?** [Perceived benefits]
2. **Now that you know the test result, how do you think it might affect your family?**
   1. Prompt – **Will it change the types of foods your family eats, how your family cooks, or the amount of exercise you/others in your family get?**
   2. **Do you think your family has health behaviors they need or want to change?**
   3. (if yes) **Could you say more about what you’d like to change?**
   4. (if yes) **What might get in the way of your family changing their behaviors?**
   5. (if no) **Could you tell me more about that?**
   6. **Do you think this test result will make your decisions about possible treatments for your child’s weight easier or harder?** [Perceived barriers]
3. **What helped you decide to have your child tested for a genetic cause of weight?**
   1. Prompt – **Going back to what you originally shared about deciding to get your child tested, was there anything else that played into your decision to do this?**
   2. **What other factors played into your decision?**
   3. Prompt **– Like the ideas of others in your family, things you have read or heard about in the news, or other things?** [Cue to action]
4. **Can you give an example of a time when you changed something in your family to make an improvement? It does not have to be related to health or weight.** Give example as needed: Implemented a homework schedule, created a sticker chart for behavior or potty training, etc.
   1. **How did it go? Did you think it was successful?**
   2. (If yes) **What do you think helped to contributed to the success?**
   3. (If no) **What got in your and your family’s way of it being successful?**
   4. (If partly or yes/no) **For the part that was successful, what do you think helped with that? For the part that was not successful, what got in the way?**
   5. **How might these experiences help or hurt your ability to make a change for your family after learning about your child’s test results?** [Self-efficacy]

**We’ve now finished the interview. Is there anything else that you want to tell us that we haven’t already talked about or anything you’ve realized throughout the interview that you’d like to share now?**

**We want to thank you for taking the time to talk to us about your family and your child today. We appreciate everything you have shared with us. If you have any questions please let us know.**
